# Structural transitions in electron beam deposited Co–carbonyl suspended nanowires at high electrical current densities

**DOI:** 10.3762/bjnano.6.134

**Published:** 2015-06-11

**Authors:** Gian Carlo Gazzadi, Stefano Frabboni

**Affiliations:** 1 S3 Center, Nanoscience Institute - CNR, Via Campi 213/a, 41125 Modena, Italy; 2 FIM Department, University of Modena and Reggio Emilia, Via Campi 213/a, 41125 Modena, Italy

**Keywords:** cobalt, electromigration, focused electron beam induced deposition (FEBID), metallic nanowires

## Abstract

Suspended nanowires (SNWs) have been deposited from Co–carbonyl precursor (Co_2_(CO)_8_) by focused electron beam induced deposition (FEBID). The SNWs dimensions are about 30–50 nm in diameter and 600–850 nm in length. The as-deposited material has a nanogranular structure of mixed face-centered cubic (FCC) and hexagonal close-packed (HCP) Co phases, and a composition of 80 atom % Co, 15 atom % O and 5 atom % C, as revealed by transmission electron microscopy (TEM) analysis and by energy-dispersive X-ray (EDX) spectroscopy, respectively. Current (*I*)–voltage (*V*) measurements with current densities up to 10^7^ A/cm^2^ determine different structural transitions in the SNWs, depending on the *I*–*V* history. A single measurement with a sudden current burst leads to a polycrystalline FCC Co structure extended over the whole wire. Repeated measurements at increasing currents produce wires with a split structure: one half is polycrystalline FCC Co and the other half is graphitized C. The breakdown current density is found at 2.1 × 10^7^ A/cm^2^. The role played by resistive heating and electromigration in these transitions is discussed.

## Introduction

The growing importance of nanotechnology and nanoscience in advanced applications and fundamental research requires nanofabrication techniques that are highly resolved but at the same time flexible and feasible with research laboratory equipment. A promising approach is represented by focused electron beam induced deposition (FEBID), a direct-write nanolithography based on the decomposition of gas precursors molecules with electron beams [1]. This technology, in fact, allows for the deposition of various materials on planar and non-planar substrates with nanoscale resolution [2], and it can be easily implemented on scanning electron microscopes (SEM) by installing a gas injection system (GIS).

FEBID flexibility has been exploited in applications that are critical for traditional lithography techniques, such as the deposition of electrical connections to isolated nanostructures [3–4] or the fabrication of scanning probe nanotips [5–6], but it has been also employed successfully in the realization of different types of nanosensors [7–10] and nanodevices [11–12]. Among the FEBID capabilities, the deposition of nanowires with nanoscale site specificity [10,13] becomes appealing for the development of future nanoscale devices which need scaled interconnects, and the deposition of magnetic nanostructures opens interesting perspectives in the field of magnetic nanodevices [14–15].

To keep up with such challenging tasks, FEBID has to face a deposit purity issue [16], the C and O contamination of metal deposits coming from incomplete fragmentation of the metalorganic molecules, typically employed as precursors. Several methods have been investigated, mainly consisting in the deposit treatment by thermal annealing [17–18] or e-beam irradiation [19], but also the design and synthesis of new precursors is considered [20].

Another important aspect, often not considered in the literature, is to investigate the electrical behavior and stability of FEBID nanodeposits under critical conditions that may occur in real devices, such as extended voltage and current ranges and high current density, where Joule heating and electromigration effects [21–22] come into play and are a major cause of failures.

In this work, we deposit free-standing suspended nanowires (SNWs) using Co–carbonyl precursor (Co_2_(CO)_8_), and study their behavior under high electrical current density, following the same approach used for Pt–metallorganic SNWs [23]. While FEBID deposits are usually grown on a substrate, suspended deposition is obtained by moving the electron beam away from an elevated edge under gas flow. If the scanning speed (beam stepsize/beam dwell time) is properly tuned, a self-standing nanowire can be deposited along the beam path [24]. This approach offers the possibility to deposit and analyze the material free from any substrate contribution, but above all it enables 3D nanofabrication [25]. The SNWs are characterized electrically at high current densities and analyzed structurally by transmission electron microscopy (TEM). The Co–carbonyl precursor has been chosen because it is one of the most commonly used for the deposition of magnetic nanostructures, and also because it yields one of the highest metal concentrations among metalorganics [26].

## Experimental

FEBID was performed in a dual beam system (FEI Strata DB235M) combining a Ga-ion focused ion beam (FIB) with a thermal field emission SEM, equipped with a Co–carbonyl (Co_2_(CO)_8_) GIS operated at room temperature (RT). The GIS is mounted at a polar angle of 52° and an azimuthal angle of 115° with respect to the sample surface. An injection nozzle with a reduced diameter of 160 μm was installed in order to limit the pressure bursts that typically occur for this kind of precursor at the first openings, and to reach a gas pressure into the chamber of the order of 3 × 10^−6^ mbar (with respect to a base pressure of 6 × 10^−7^ mbar), a value that allowed for a fine control of the deposition process. The nozzle-to-sample distance during deposition was about 200 μm.

The samples are Au-coated (100 nm thickness) silicon nitride membranes (500 nm thick). Pairs of contact pads were patterned on gold by FIB milling, and, at the gap between the pads, a slit (500 nm wide and 6 μm long) was opened through the membrane to enable TEM observation and obtain substrate-less suspended growth. SNWs were deposited across the slit by focusing a 15 keV, 67 pA electron beam (probe size about 5 nm) either on Co nanopillars, grown by FEBID, or directly on the gold pads, and moving it towards the opposite side with 5 nm steps and dwell times varying between 10 and 35 ms in order to obtain the desired horizontal growth.

Electrical characterization was done in situ using two nanomanipulated probes (Kleindiek MM3A-EM) connected to a Keithley 6487 source meter. The current (*I)*–voltage (*V*) measurements were performed by applying voltage to the left Au pad and sweeping it over a (0, +*V*, −*V*, 0) loop with the right pad grounded (GND) while measuring the current. After each *I*–*V* curve an SEM image of the SNW was taken to check for morphological modifications. TEM analysis was performed with a JEOL 2010 microscope, equipped with energy dispersive X-ray spectroscopy (EDX) system (Oxford INCA 100) for composition analysis.

## Results and Discussion

**Figure 1:**
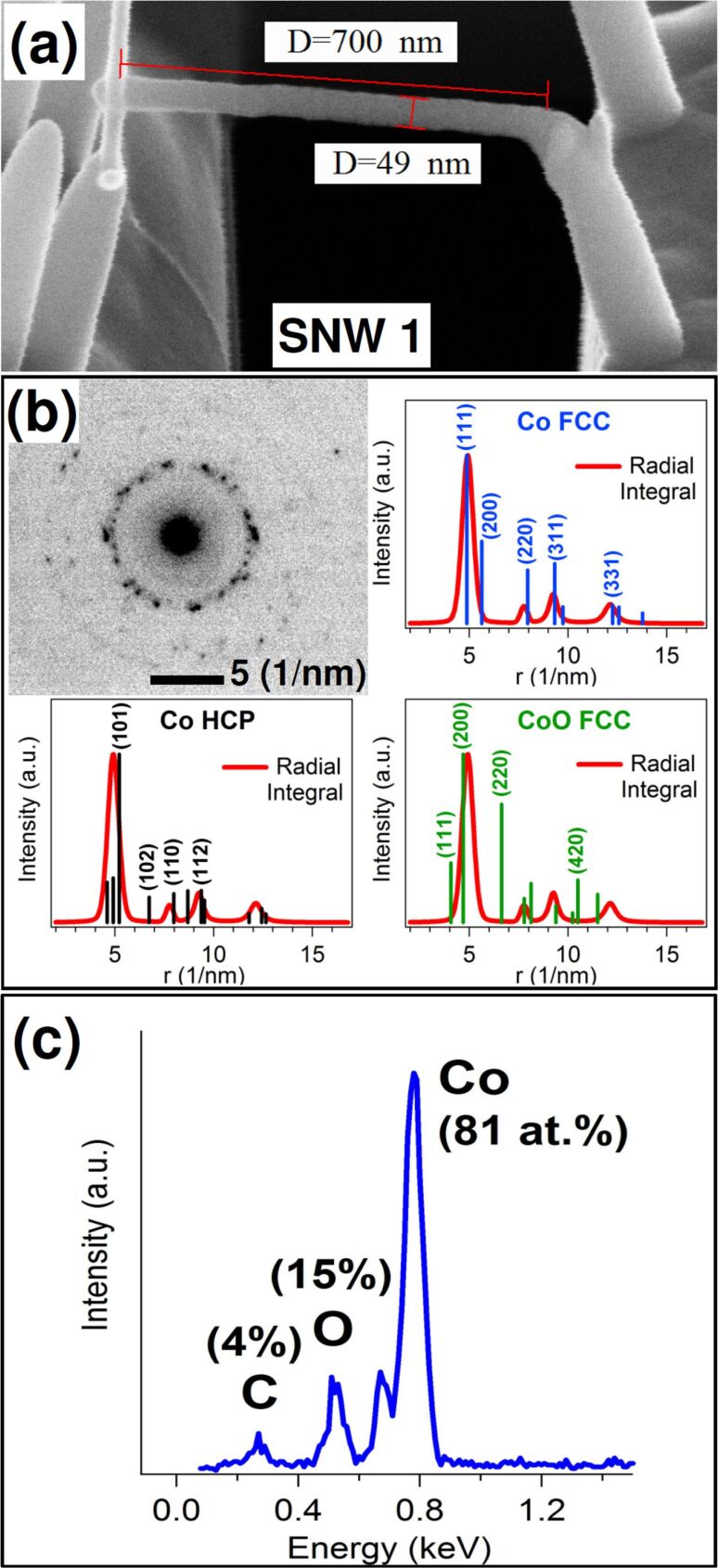
(a) SEM image (at 52° tilt angle) of suspended nanowire (SNW) 1 deposited between pillars; (b) electron diffraction pattern from SNW 1 and radial integral of the pattern (red line) compared with calculated reflections (bars) for FCC Co, HCP Co and FCC CoO. (c) EDX spectrum of SNW 1 with peak labels and derived atomic composition.

In Figure 1a, an example of Co–carbonyl SNW deposited by FEBID is shown. The suspended wire is deposited across the slit and connects two Co–carbonyl nanopillars facing on the opposite sides. Deposition of the SNW is performed by focusing the beam, normal to the sample, on the right pillar and moving it toward the left with the scan parameters specified before. The obtained SNW (SNW 1) is about 700 nm long, 50 nm thick and 30 nm wide, and because it is slightly sloped upward another deposition (30 nm wide and 65 nm long) was necessary to connect to the left pillar. The deposited material shows a uniform bright contrast under the SEM, and TEM imaging (not shown) reveals a nanogranular structure typical of metallorganic deposits, where metal grains with a size of few nanometers are embedded in an amorphous, carbonaceous matrix [27]. The structure is confirmed by TEM selected area electron diffraction (SAED) measured at the center of the wire and presented in Figure 1b. The pattern shows an innermost high-intensity ring with many single spots, and outer, fainter rings with spots apparently randomly arranged. This kind of pattern is typical of nanocrystalline materials with randomly oriented nanograins. To establish the structure of these nanograins the radial integral of the pattern (red curve) was compared to the main reflections calculated for face-centered cubic (FCC) Co, hexagonal close-packed (HCP) Co and FCC CoO, displayed as bars proportional to the intensities of the reflections. The experimental curve shows the main peak at *r* = 4.9 1/nm and three smaller structures at *r* = 7.7, 9.2 and 12.1 1/nm. The best agreement is found with the Co FCC pattern, which can fit all the four peaks with (111), (220), (311) and (331) reflections, respectively. A lower degree of agreement also exists with the Co HCP pattern. On the contrary, the absence of any structure at *r* = 6.6 and 4.0 1/nm, corresponding to the second (220) and third (111) most intense reflections of CoO FCC, suggests that such a phase is not present. This comparison indicates that the deposited material is a mixture of FCC (a larger fraction) and HCP cobalt nanograins. EDX analysis was also performed by TEM. The measured spectrum, in Figure 1c, shows the peaks of the precursor components, C, O and Co, with atomic concentrations of 4, 15 and 81 atom %, respectively. This high Co concentration is in line with the best values reported in the literature for this precursor [25], though concentrations above 90 atom % have also been obtained [10,26,28].

**Figure 2:**
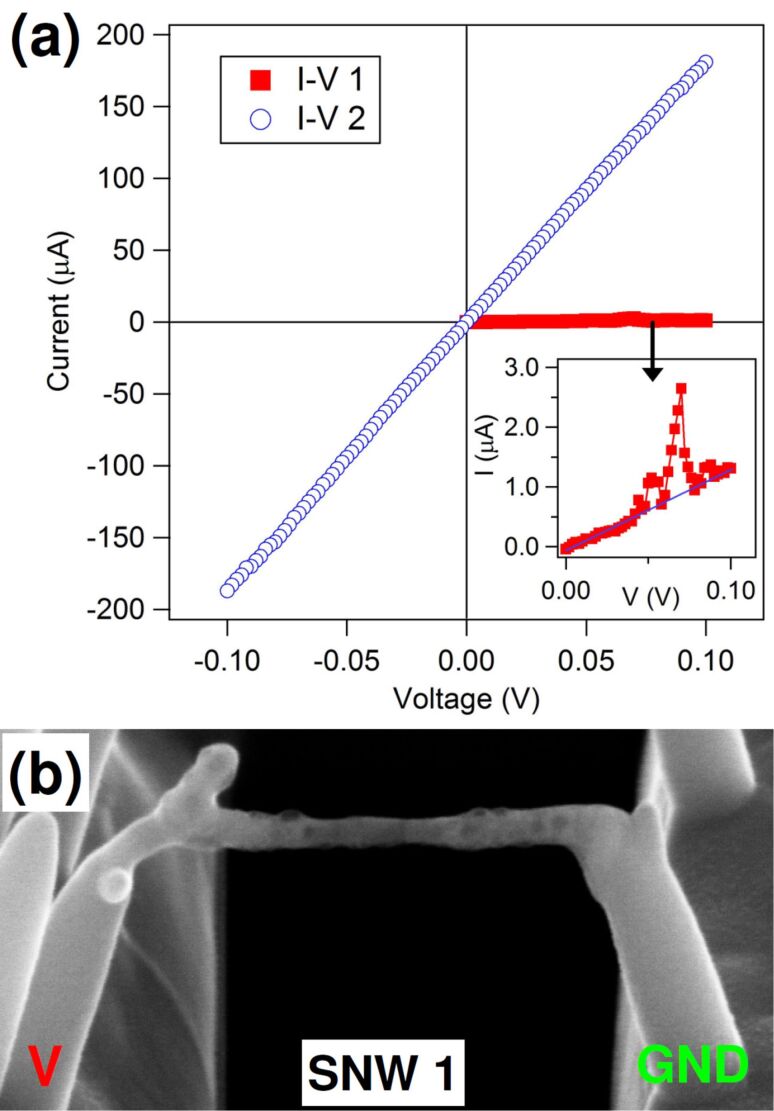
(a) Current(*I*)–voltage(*V*) measurements on SNW 1. In the inset, the first *I*–*V* measurement taken on the wire as shown in Figure 1a is magnified; (b) SEM image (at 52° tilt angle) of SNW 1 after *I*–*V* 1.

Electrical characterization of SNW 1, shown in Figure 2a, was carried out inside the dual beam system. The bias range in this case was *V* = 0.1 V. In the first measurement, shown in the inset of Figure 2a (squares), the current starts increasing linearly with voltage, but around *V* = 0.07 V, a big spike from *I* = 0.7 to 2.7 μA is recorded, followed by a return to values on the original linear trend. The measurement was stopped before finishing the loop to observe SNW 1. The SEM image is shown in Figure 2b. It is clear that a structural transformation has occurred due to the current spike, because the nanowire shows a completely changed morphology, with dark bumps and spots on the surface and inside, and a reduced thickness in the middle. The connection to the left pillar is heavily bent and deformed as if a mechanical stress was applied. The supporting pillars do not show such modifications and maintain the same uniform bright contrast. A second *I*–*V* measurement was taken on the same bias range, completing the cycle, and data are shown in Figure 2a (circles). The *I*–*V* trend is strictly linear, and current values are increased dramatically from the previous run, reaching almost *I* = 200 μA at *V* = 0.1 V. The resistivity obtained considering SNW 1 alone drops from 1.6 × 10^4^ μΩ·cm, before the transition, to about 110 μΩ·cm, a value to be compared to 6 μΩ·cm for bulk cobalt. SEM inspection after this measurement shows no further difference from the picture in Figure 2b.

**Figure 3:**
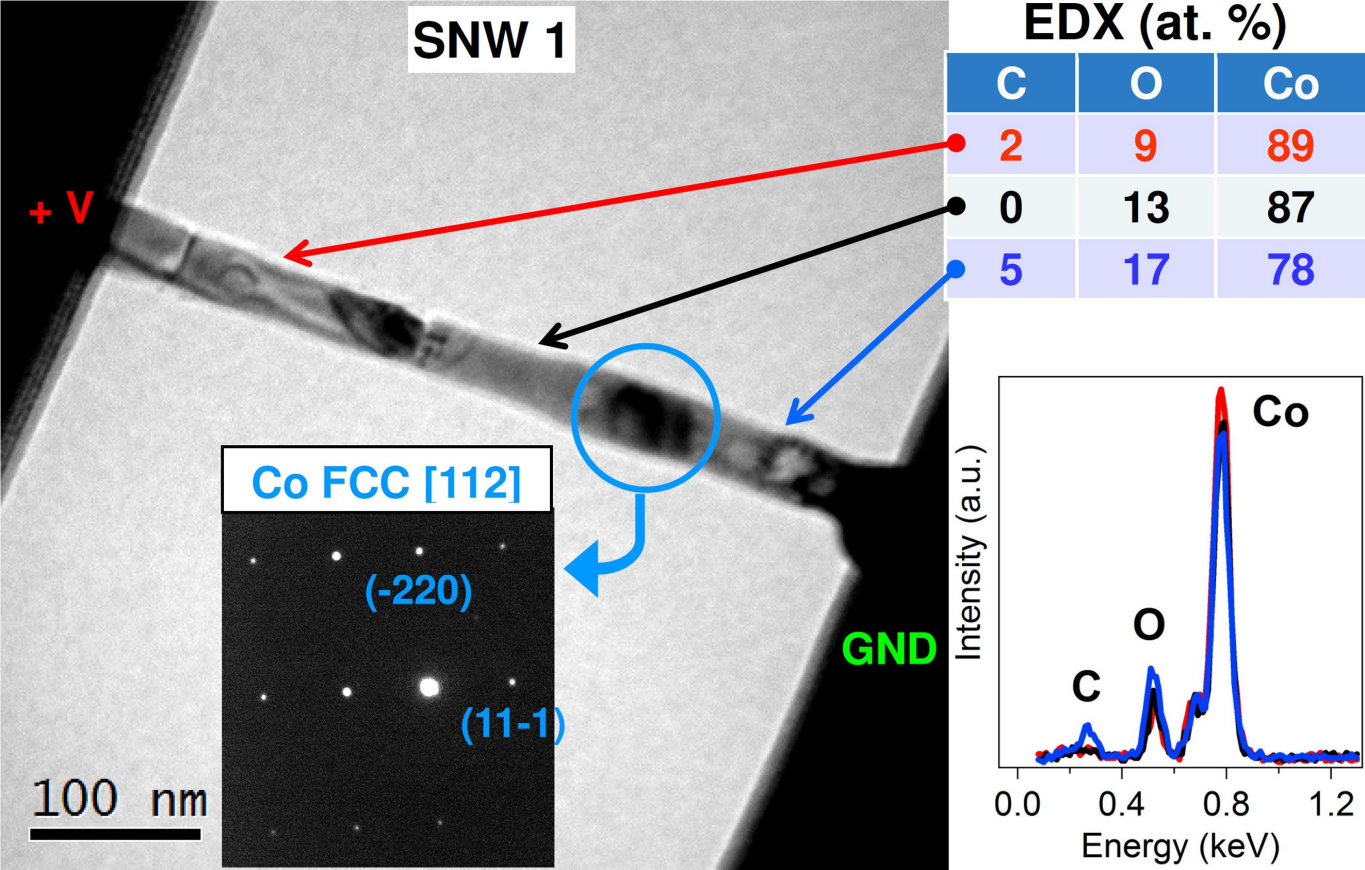
Bright-field TEM image of SNW 1 after electrical measurements. In the inset, the SAED pattern taken on the circled area, with labelled phase and spots. On the right, the EDX spectra and a table of the relative atomic compositions taken along SNW 1 in three points indicated by the arrows.

TEM structural analysis after these electrical measurements is reported in Figure 3. The bright-field image of SNW 1 is completely changed from the grainy structure observed after deposition. Now it has the typical appearance of a polycrystalline material, with regions of well-defined contrast extending for tens of nanometers along the wire, and separated by sharp contrast lines. This suggests the presence of big metal grains inside the nanowire. The SAED pattern taken on the central dark region (blue circled), 35 nm wide and 55 nm long, shows single spots arranged in a rectangular lattice that correspond to the FCC structure of Cobalt, oriented along the [112] zone axis. This type of pattern indicates that a highly ordered, essentially monocrystalline, structure is present within this region. Other orientations of the same Co FCC phase were found along the wire, while the bright circles on the right turn out to be hollow graphite cages, as will be shown for the second SNW. EDX analysis performed along the wire reveals a small gradient in Co concentration on going from right to left: 78 atom % on the right, 87 atom % on the center and 89 atom % on the left.

Interestingly, this distribution follows the electron current direction, from cathode (right) to anode (left), suggesting that an electromigration effect is involved in the structural transition. This effect occurs in metallic micro- and nanowires under high current densities (10^6^ to 10^7^ A/cm^2^) and consists in the dragging of metal ions along the electron current direction due to momentum transfer by the flowing electrons [21]. From EDX spectra, shown in the right-bottom panel of Figure 3, it is also evident that a true carbon peak, above background level, remains only on the right part of SNW 1, where the graphite circles were observed.

**Figure 4:**
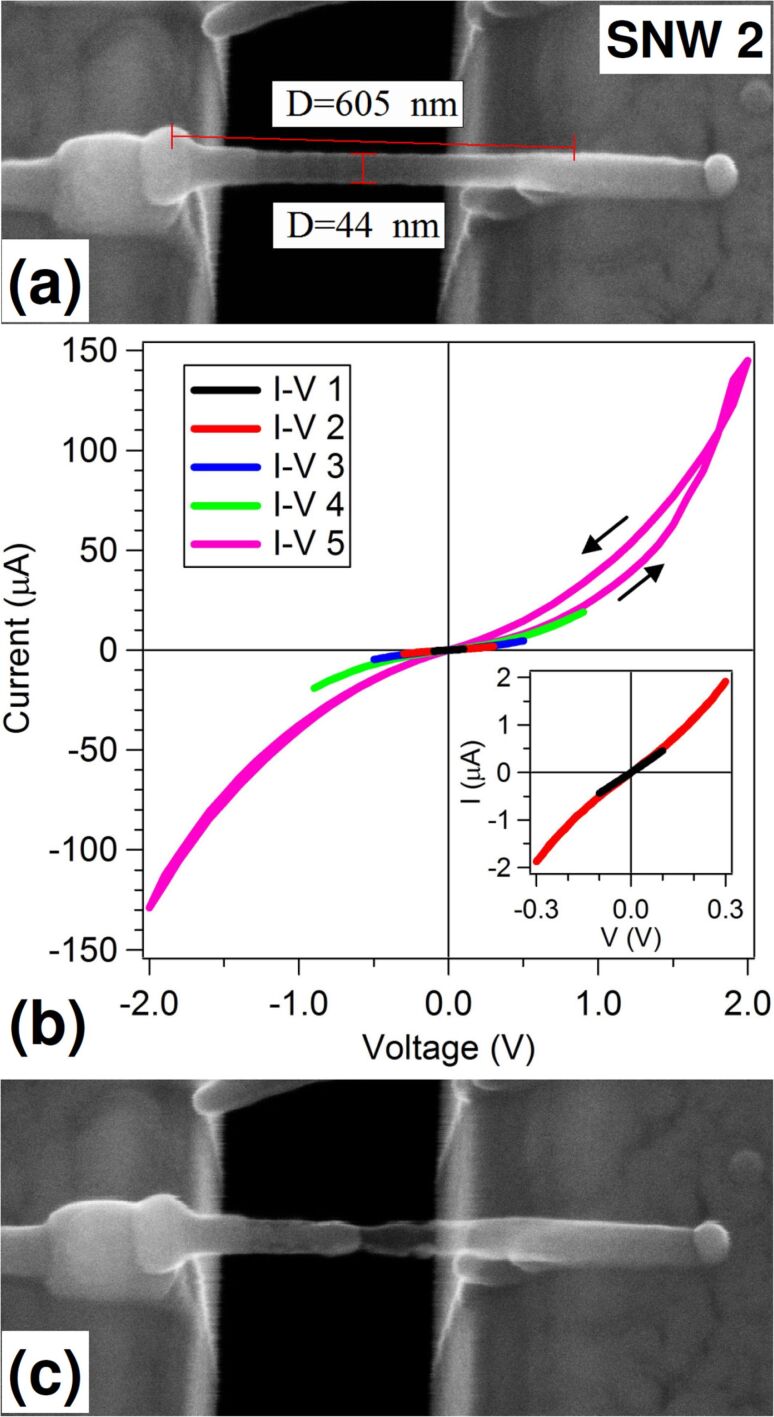
(a) SEM image (at 20° tilt angle) of SNW 2 deposited between the Au pads across the slit. (b) Five subsequent *I*–*V* measurements taken on SNW 2 at increasing bias range. In the inset, the first two *I*–*V* curves are shown; (c) SEM image (at 20° tilt angle) of SNW 2 after the last *I*–*V* measurement.

A second SNW (SNW 2), shown in Figure 4a, was deposited directly between the Au contacts without pillars. It is about 600 nm long, 45 nm thick and 30 nm wide. To ensure good connection to the left pad, an additional deposition was performed on a square area around SNW end. EDX analysis, not shown, returned concentrations similar to SNW 1: 5 atom % C, 17 atom % O and 78 atom % Co. SNW 2 was tested with five subsequent *I*–*V* measurements, shown in Figure 4b, extending the voltage range from 0.1 to 2 V, and after each one of them a check for morphology changes was done by SEM. The first measurement, shown in the inset of Figure 4b (black line), has a linear behavior with a resistance of 223 kΩ and resistivity of 5 × 10^4^ μΩ·cm, while the second *I*–*V* curve (red line) shows a slight upward bending as the voltage increases above 0.1 V. All subsequent *I*–*V* curves show an increasing bending for increasing voltage. These semiconductor-like trends are typical of potential-barrier conduction systems, such are the nanogranular FEBID deposits, but they might also include an Au/SNW contact barrier that the two probe setup is not able to cancel. SEM observation after the first four cycles did not reveal any modification in SNW 2. In the last *I*–*V* curve (pink line), a different behavior appears: an hysteresis between [0,2 V] and [2,0 V] data is present, the return curve having higher currents with respect to the first leg. The negative bias portions reflect the return curve trend and do not show any hysteresis. The observed effect might be linked to some structural transformation that was indeed confirmed by SEM analysis, in Figure 4c. The left-hand half of the wire looks brighter while the right-hand one has become transparent.

**Figure 5:**
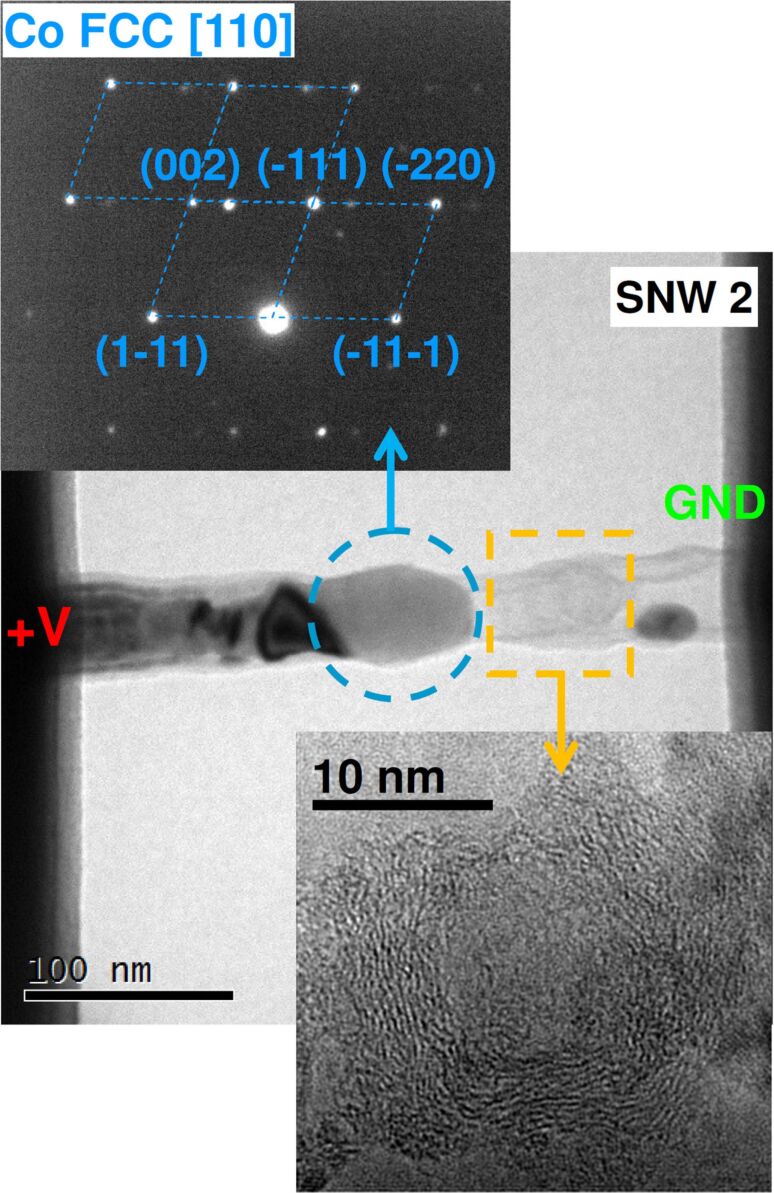
Bright-field TEM image of SNW 2 as shown in Figure 4c. In the top inset, the SAED pattern taken on the blue-circled area, with labelled phase and spots. In the bottom inset, the high-resolution image of the orange-squared area.

To deeper investigate the nature of this transition we turned to TEM analysis. As shown by the bright-field image in Figure 5, the opaque portion on the left is polycrystalline cobalt while the transparent region on the right is graphitized carbon. The SAED pattern taken on the big central grain (55 nm wide and 65 nm long, blue circled), after a tilt of the sample, is shown in the upper panel. The spots arranged in a diamond lattice reveal that the grain has an FCC structure oriented along the [110] zone axis.

The extra spots around the main lattice arise from nearby grains entering the diffraction volume due to the sample tilt. A high-resolution image of the transparent region (orange squared) is reported in the lower panel, and shows graphite planes arranged in a rounded cage structure with a hollow/amorphous-like interior. This carbon structure, strongly resembling the one of carbon-encapsulated metal nanoparticles [29], was probably hosting a Co grain before its migration. From this analysis, the effect of electromigration is even more evident than before: the metallic part has all migrated toward the anode, leaving a graphitic skeleton behind.

**Figure 6:**
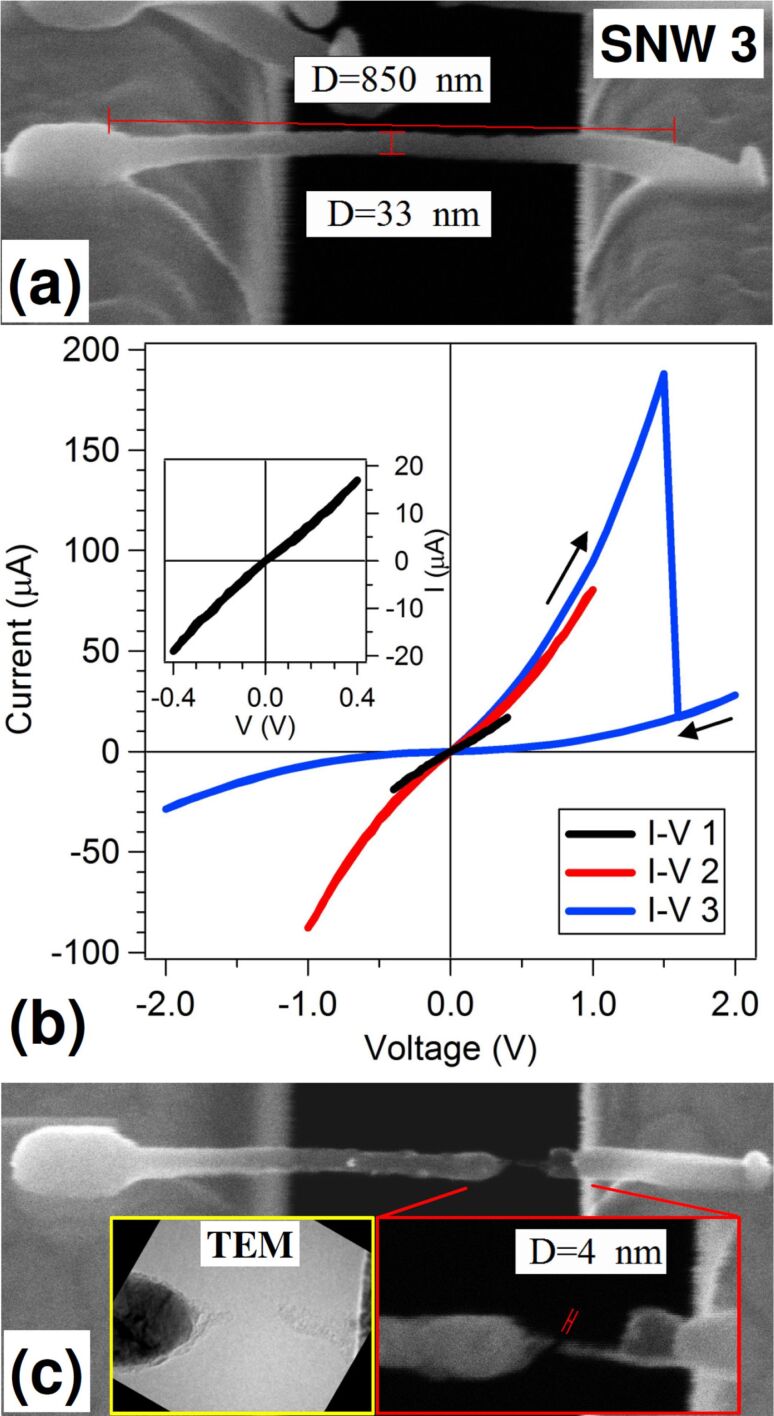
(a) SEM image (at 52° tilt angle) of SNW 3 deposited between Au pads; (b) Three subsequent *I*–*V* measurements on SNW 3 at increasing bias range. In the inset, the first *I*–*V* curve is shown; (c) SEM image (at 20° tilt angle) of SNW 3 after the last *I*–*V* measurement. In the bottom-right and bottom-left insets, a magnified view of the gap region taken with the SEM (at 52° tilt angle) and with the TEM, respectively, are shown.

Finally, a third SNW (SNW 3) was deposited between the Au pads and stressed electrically until breakdown. As shown in Figure 6a, SNW 3 is 850 nm long, 30–35 nm thick and 25–30 nm wide. A square deposition on SNW end was applied as for SNW 2. EDX analysis, not shown, gave concentrations of 6 atom % C, 13 atom % O and 81 atom % Co. The first *I*–*V* measurement, shown in the inset of Figure 6b, was taken with a bias of *V* = 0.4 V and displays a linear behavior, slightly deviating at the extreme points of the negative interval. The measured resistance is 23 kΩ, corresponding to a resistivity of 2.5 × 10^3^ μΩ·cm. The second measurement (red line) starts exhibiting the bent-up characteristic, less pronounced than for SNW 2, and also an asymmetry between positive and negative branches. At *V* = −1 V, in fact, a 10% increase in the absolute current is recorded, possibly indicating that structural modifications are going on during measurement. The third and last *I*–*V* curve (blue line) rises up steeper and at *V* = 1.5 V, *I* = 190 μA, corresponding to a current density of 2.1 × 10^7^ A/cm^2^, it drops down not to zero but to *I* = 17 μA, where it keeps following a bent-up trend to 2 V and finishes the cycle drawing a symmetric negative branch. The SEM image taken at the end of the measurement (Figure 6c) clearly shows a gap in the SNW with Co depletion in the terminal of the right section, which appears transparent.

By zooming in the gap region (bottom-right inset) we can distinguish a very narrow gap (4 nm) separating the left part, ending with a dark tip on a bright grain, and the right part, ending with a 9 nm thick, 45 nm long dark, i.e., transparent tip. TEM analysis (bottom-left inset) confirms that the right section is polycrystalline Co ending with a tiny tip of graphitized C, and the tip on the right is also graphitized C. A question arises whether the current measured after the drop is flowing through a still-continuous C bridge or it is tunneling across the gap. The second case seems to be excluded because the current values are too high and the curve does not fit a Fowler–Nordheim model. The first option is favored because the cross section reduction from 35 to 9 nm diameter, observed at the bridge, can roughly account for a current drop of a factor of 13. If this is the case, then the gap probably formed after measurement by some vibration during nanoprobes lift-up.

At the basis of the structural evolution observed in the three SNWs there is an interplay of several factors. Primarily the physical effects related to high current density (Joule heating and electromigration), but also geometrical factors represented by the suspended geometry and nanosize dimension of the conductor, and the highly-resistive nanogranular material. Let us first consider the materials obtained at the end of the electrical measurements: polycrystalline FCC cobalt and graphitized carbon. The equilibrium phase of bulk cobalt at RT is HCP, and at 430 °C there is a transition to the FCC phase. Co is known to stabilize FCC at RT as a consequence of either rapid quenching from annealing above the transition temperature or of the grain size confinement to submicron range [30]. The transition temperature is easily surpassed during the electrical measurements, where current densities up to 10^7^ A/cm^2^ are injected into the SWNs. As already shown in our previous study on Pt SNWs [23], and in W nanowires either suspended [31] or deposited on ultrathin membranes [32], the temperature reached in such conditions can be as high as or exceed 1000 °C. The fact that cobalt does not stabilizes back into the HCP phase at the end of the measurement, when the SNWs return to RT, is a consequence of the nanosize cross-section of the wire: FCC structure at RT is often reported for both Co nanoparticles and nanowires in the diameter range of tens of nanometers [33]. Concerning graphitized carbon, this material is formed around the Co grains, acting as catalyzers, under the combined action of high temperature and nanograin motion, as reported in similar amorphous C nanowires loaded with Fe nanoparticles [34].

A second consideration regards the distribution of cobalt along the wires and its connection to *I*–*V* measurement history and electromigration. In SNW 1, the Joule heating during the current burst is making the wire fully metallic and polycrystalline. The current density associated to the burst (1.8 × 10^5^ A/cm^2^) is relatively low for electromigration to be effective, and during the second measurement, though a much higher current density is reached (1.3 × 10^7^ A/cm^2^), the electromigration effect is only minor (a small Co concentration gradient) because Co ions are strongly bound in large monocrystalline grains. In SNW 2, the current density increases progressively, reaching 10^7^ A/cm^2^ in the last cycle, where the SNW structure is still apparently unchanged from the as-deposited one. So this high electron current is flowing through a nanogranular material and can more easily displace and accumulate the Co ions toward the anode side. The gap morphology observed in SNW 3 may have a twofold interpretation. It could be considered as an evolution of the final SNW 2 structure, where, at the boundary between Co grain and graphitized C, a neck forms as the current density is increased, and the C portion thins down to form a bridge of a few nanometers. Alternatively, looking at the shape of SNW 3 at the bridge (bottom-right inset of Figure 6c), the sharp SNW truncation on the right could suggest that a big Co grain was present above the bridge and has moved left, leaving a void.

## Conclusion

In conclusion, we have deposited Co (80 atom % concentration) SNWs from Co–carbonyl precursor by FEBID, and characterized them electrically at increasing bias range to reach high current density (10^7^ A/cm^2^). Starting from a nanogranular material of mixed FCC and HCP Co grains, Joule heating leads to the formation of polycrystalline FCC Co already at low voltages and current densities. Different structural morphologies are observed in the SNWs depending on the *I*–*V* history. With a short current burst the wire becomes fully polycrystalline Co and shows ohmic behaviour. When repeated *I*–*V* measurements at increasing voltage are performed, an electromigration effect becomes dominant dividing the wire in two halves: a metallic portion, on the anode side, and a graphitic carbon portion on the cathode side. The highest current density reached before breakdown is 2 × 10^7^ A/cm^2^.
